# Association of Body Mass Index and Plant-Based Diet with Cognitive Impairment among Older Chinese Adults: A Prospective, Nationwide Cohort Study

**DOI:** 10.3390/nu14153132

**Published:** 2022-07-29

**Authors:** Fang Liang, Jialin Fu, Gabrielle Turner-McGrievy, Yechuang Wang, Nan Qiu, Kai Ding, Jing Zeng, Justin B. Moore, Rui Li

**Affiliations:** 1 School of Public Health, Wuhan University, Wuhan 430071, China; fliang@whu.edu.cn (F.L.); fjl0708@whu.edu.cn (J.F.); ywang20@whu.edu.cn (Y.W.); 2013302170051@whu.edu.cn (N.Q.); 2021203050024@whu.edu.cn (K.D.); 2021283050065@whu.edu.cn (J.Z.); 2 Department of Health Promotion, Education, and Behavior, Arnold School of Public Health, University of South Carolina, Columbia, SC 29208, USA; brie@sc.edu; 3 Department of Implementation Science, Division of Public Health Sciences, Wake Forest School of Medicine, Winston-Salem, NC 27101, USA; jusmoore@wakehealth.edu; 4 School of Nursing, Wuhan University, Wuhan 430071, China

**Keywords:** cognitive impairment, body mass index, plant-based dietary pattern, older Chinese adults, cohort

## Abstract

To examine the association of body mass index (BMI) and a plant-based diet (PBD) with cognitive impairment in older adults, this cohort study used data from the Chinese Longitudinal Healthy Longevity Survey (CLHLS), a national, community-based, longitudinal, prospective study in China. Cognitive function was evaluated via the Mini-Mental State Examination (MMSE). Diet was assessed using a simplified food frequency questionnaire (FFQ), and PBD patterns were estimated using the overall plant-based diet index (PDI), the healthful plant-based diet index (hPDI), and the unhealthful plant-based diet index (uPDI). BMI was measured objectively during the physical examination. Cox proportional hazard models and restricted cubic spline analyses were used. A total of 4792 participants with normal cognition at baseline were included, and 1077 participants were identified as having developed cognitive impairment during the 24,156 person-years of follow-up. A reverse J-shaped association was observed between BMI and cognitive impairment (*p* = 0.005 for nonlinearity). Participants who were overweight (HR = 0.79; 95% CI 0.66–0.95) and obese (HR = 0.72; 95% CI 0.54–0.96) had a decreased risk of cognitive impairment, while those who were underweight (HR = 1.42; 95% CI 1.21–1.66) had an increased risk. Lower PDI, lower hPDI, and higher uPDI were associated with an increased risk of cognitive impairment (HR = 1.32; 95% CI 1.16–1.50 for PDI; HR = 1.46; 95% CI 1.29–1.66 for hPDI; HR = 1.21; 95% CI 1.06–1.38 for uPDI). The protective effect of being overweight on cognitive impairment was more pronounced among participants with a higher PDI (HR = 0.74; 95% CI 0.57–0.95) than those with a lower PDI (HR = 0.87; 95% CI 0.67–1.12), among participants with a higher hPDI (HR = 0.73; 95% CI 0.57–0.94) than those with a lower hPDI (HR = 0.93; 95% CI 0.72–1.10), and among participants with a lower uPDI (HR = 0.61; 95% CI 0.46–0.80) than those with a higher uPDI (HR = 1.01; 95% CI 0.80–1.27). Our results support the positive associations of overweight status, obesity, an overall PBD, and a healthful PBD with cognitive function in older adults. A lower adherence to an overall PBD, a healthful PBD, and a higher adherence to an unhealthful PBD may attenuate the protective effect of being overweight on cognitive function.

## 1. Introduction

With the global population ageing, the number of older adults with dementia is set to rise substantially across the world. Nearly 46 million individuals were affected by dementia worldwide in 2015, and that number is predicted to reach 152 million in 2050 [1]. Dementia is a common and serious neurodegenerative disorder of older adults which impairs quality of later life and imposes a heavy burden on the affected individuals, their families, and the economy [2]. As there are currently no effective treatments for dementia, prevention is of major importance in fighting this disease [3]. Cognitive impairment is a prodromal phase of dementia that provides an opportunity to take steps to prevent dementia [4]. Therefore, the recognition of possibly modifiable risk factors for cognitive impairment is of great importance for dementia prevention.

Increasing attention has been paid to associations between weight status, measured by body mass index (BMI), and cognitive function in older populations. Although the mechanism has not been completely explained, it has been widely proposed that unfavorable weight status may affect metabolic functions, promote inflammation, and disrupt the balance of gut microbiota, which could increase the risk of poor cognitive function [5]. However, previous epidemiological studies have shown conflicting results between BMI and cognitive function, with some research suggesting that higher BMI contributes to poor cognitive function [6,7,8,9,10,11,12], and other studies observing an apparent beneficial effect of higher BMI on cognitive function [13,14,15,16,17,18,19,20,21]. Also, few large, prospective cohort studies have been conducted in the older Chinese population. Our previous results suggested that a larger BMI and a BMI-defined overweight status were related to slower cognitive decline [13]. A cohort study reported that BMI-defined overweight status was associated with a lower risk of cognitive impairment [22], and another recent cohort study suggested that a BMI-defined underweight status was related to a higher risk of cognitive impairment [23].

Plant-based foods are a rich source of antioxidants and anti-inflammatory nutrients that could reduce inflammation and oxidative stress in the central nervous system [24,25,26]. Several studies have linked plant-based diets (PBDs), which are characterized by a higher consumption of plant-based foods and a lower or no intake of animal-based foods, with better neurological health [26,27]. However, previous studies on PBDs are somewhat limited because due to the lack of differentiation between the quality of plant-based foods. Recent research further defined three plant-based diet indices (PDIs), including the overall plant-based diet index (PDI), the healthful plant-based diet index (hPDI), and the unhealthful plant-based diet index (uPDI), so as to consider the dietary quality of a PBD. For instance, the PDI assesses alignment with diets higher in plant-based foods and lower in animal-based foods, the hPDI emphasizes a high consumption of healthful plant-based foods and a low consumption of unhealthful plant-based foods, and the uPDI is the opposite of the hPDI in that it emphasizes a high consumption of unhealthful plant-based foods within the context of an overall PBD [28,29,30]. Previous research has shown that healthful plant-based foods (e.g., fresh vegetables and fresh fruits) were related to better neurological health, while unhealthful plant-based foods (e.g., preserved vegetables and added sugars) were related to poor neurological health [27,31]. To date, relatively little research has investigated the relationship between plant-based dietary patterns (overall PBD, healthful PBD, and unhealthful PBD) and cognitive function [32,33].

Currently, the evidence for a potential moderating role of a PBD in the relationship between BMI and cognitive function is scarce. To fill this knowledge gap, we utilized a nationally representative sample of older Chinese adults to prospectively evaluate the association of BMI with cognitive impairment, explore the associations of three plant-based dietary patterns with cognitive impairment, and examine the potential moderating role of a PBD in the association between BMI and cognitive impairment.

## 2. Methods

### 2.1. Study Population

As detailed elsewhere [34,35], the Chinese Longitudinal Healthy Longevity Survey (CLHLS) is an ongoing, prospective cohort study among Chinese adults aged 65 years and older that was established in 1998 using multistage cluster sampling, and recruiting participants from 23 out of the 31 provinces in China, thus covering about 85% of the total population in China. Follow-up surveys were conducted every 3 or 4 years. All participants signed written informed consent for the baseline and follow-up surveys. The CLHLS study was approved by the Biomedical Ethics Committee of Peking University, Beijing, China (IRB00001052–13074).

**Figure 1:**
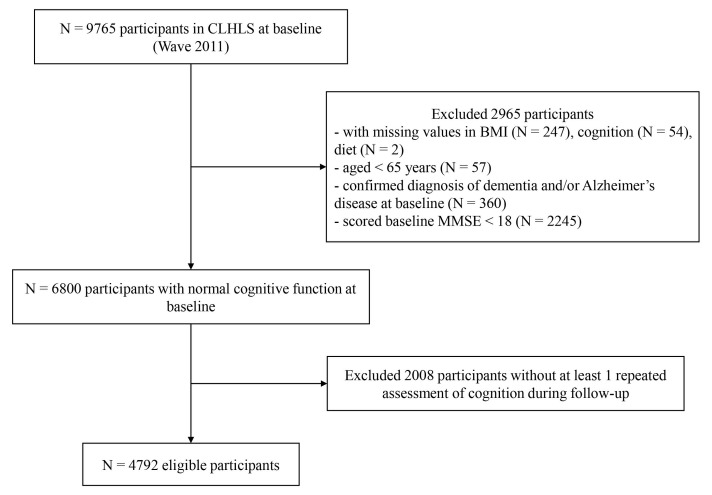
Flow chart of participants.

Since the height and weight information were first objectively measured in the sixth wave (2011), our research considered the sixth wave (2011) as the baseline. The seventh wave (2014) and the eighth wave (2018) were considered as the follow-up. Figure 1 shows the detailed flowchart of participant selection for the current study. A total of 9765 participants attended the 2011 cycle survey of the CLHLS. Of these, 360 were excluded for the following reasons: they had missing height or weight measurements (*n* = 247), they did not complete the cognitive measurements (*n* = 54), they did not complete the dietary assessments (*n* = 2), or they were younger than age 65 at baseline (*n* = 57). An additional 2245 participants were excluded due to cognitive impairment at baseline, and an additional 360 participants had a confirmed diagnosis of dementia and/or Alzheimer’s disease at baseline. In addition, 2008 participants without at least one follow-up assessment of cognition were excluded. The remaining 4792 individuals were included in the analyses.

### 2.2. Measurement and Calculation of Body Mass Index

Body weight (in kilograms) and height (in centimeters) were measured by trained assessors following standardized procedures. BMI, defined as the weight (kg) in kilograms divided by the height (m) squared, was categorized as: underweight (BMI < 18.5 kg/m^2^), normal (18.5 ≤ BMI < 24 kg/m^2^), overweight (24 ≤ BMI < 28 kg/m^2^) and obese (BMI ≥ 28 kg/m^2^) [21].

### 2.3. Assessment of Cognitive Function

The CLHLS used the Chinese version of the Mini-Mental State Examination (MMSE) to evaluate cognitive function. The MMSE contains a total of 30 items that assess orientation, registration, attention and calculation, recall, and language, with a score range from zero to 30 [36,37]. Use of the MMSE in the CLHLS is well-documented as both reliable and valid [22,35,38,39,40,41]. Since MMSE scores might be influenced by education level [40], participants were defined as cognitively impaired following education-based MMSE cutoff points. Specifically, we used the MMSE scores of 18, 20, and 24 as the cut-off points for subjects with no formal education, only a primary school education (1–6 years), and a middle-school or higher education (>6 years), respectively [32,40].

### 2.4. Measurement and Calculation of Plant-Based Diet Indices

Each participant’s dietary information was collected using a simplified food frequency questionnaire (FFQ). The questionnaire has been broadly used, with its reliability and validity both well-supported [28,40,42,43,44]. The simplified FFQ in the CLHLS included 16 food groups which are commonly consumed in China. In the present study, we divided the 16 food groups into 3 categories according to their potentially divergent health effects, including healthful plant-based foods (whole grains, fresh fruits, fresh vegetables, legumes, garlic, vegetable oils, nuts, and tea), unhealthful plant-based foods (refined grains, preserved vegetables, and sugar (white granulated sugar or candies)), and animal-based foods (animal fat, eggs, fish and aquatic products, meat, and milk and dairy products) [29,45,46]. For legumes; garlic; nuts; tea; salted, preserved vegetables; sugar (white granulated sugar or candies); eggs; fish; meat; and milk, the questionnaire had 5 options, including “almost every day”, “≥1 time per week”, “≥1 time per month”, “occasionally”, or “rarely or never”. For whole grains, refined grains, vegetable oil, and animal fats, the questionnaire had two options, including “yes” and “no”. For fruits and fresh vegetables, the questionnaire had four options, including “almost every day”, “quite often”, “occasionally”, or “rarely or never”.

Using this dietary data, we computed the PDI, the hPDI, and the uPDI to evaluate the overall PBD pattern, the healthful PBD pattern, and the unhealthful PBD pattern, respectively [28,29,40]. Intake frequencies of the 16 food groups were assigned a score between 1 and 5. For the PDI, plant-based food groups were given positive scores (1 for the least frequent consumption and 5 for the most frequent consumption), whereas animal-based food groups were given reverse scores (5 for the least frequent consumption and 1 for the most frequent consumption). For the hPDI, healthful plant-based foods were given positive scores, but unhealthful plant-based foods and animal-based foods were reverse scored. For the uPDI, healthful plant-based foods and animal-based foods were reverse scored, but unhealthful plant-based foods were given positive scores. We summed the 16 food-group scores for everyone to derive the PDI, hPDI, and uPDI, with a theoretical range of 16 to 80. More detailed information on calculating the PDI, hPDI, and uPDI are provided in Table A1. In the present study, the PDI, hPDI, and uPDI were classified into 2 halves based on the median level, including a lower half (lower PDI, lower hPDI, and lower uPDI) and a higher half (higher PDI, higher hPDI, and higher uPDI), respectively.

### 2.5. Assessment of Covariates

Covariates shown by prior research that could alter the associations of the BMI and a PBD with cognitive function were adjusted in our analyses. Potential confounders included age (years), sex (male or female), type of residence (city, town, or country), education (illiterate or literate), main occupation before 60, smoking status (current, former, or never), drinking status (current, former, or never), financial status (financial dependence or independence), regular exercise (yes or no), and health conditions. Health conditions were evaluated by taking into consideration six diseases: hypertension, diabetes, heart disease, stroke, cancer, and respiratory disease. Each disease was scored 1 (present) or 0 (not present).

### 2.6. Statistical Analysis

Descriptive statistics were used to summarize the baseline characteristics. Cox proportional hazard models were conducted to evaluate the association of baseline BMI with cognitive impairment using categories of BMI with the normal group as the reference. We also used Cox proportional hazard models to examine the associations of PDIs, hPDIs, and uPDIs with cognitive impairment. The follow-up period for each individual was computed from baseline to the date of the first occurrence of cognitive impairment, to the date of death, lost-to-follow-up, or to the end of follow-up, whichever occurred first. The proportional hazards assumption was verified by using a global test for zero slope of the scaled Schoenfeld residuals over time. In addition, we performed a restricted cubic spline with 4 knots placed at the 5th, 35th, 65th, and 95th percentiles, and we used the median value of the baseline BMI as a reference point to test the potential non-linear association of the baseline BMI with cognitive impairment. We performed stratified analyses by PDI, hPDI, and uPDI score to assess whether the associations of BMI and cognitive impairment varied with PDI, hPDI, and uPDI scores. The regression models included sex, age, residence, education, occupation, smoking status, drinking status, regular exercise, financial independence, and health conditions.

Data were analyzed using STATA 16 (StataCorp, College Station, TX, USA) and R software, version 3.4.2 (R Foundation, Vienna, Austria). Tests were two-sided with the statistical significance set as *p* < 0.05.

## 3. Results

Of the 4792 participants included, 2425 (50.61%) were men, and there was a mean age of 80.70 ± 9.58 years old at baseline. In total, 2493 (52.02%) participants were living in rural locations, 2339 (48.81%) were illiterate, 2972 (62.02%) were never smokers, and 3133 (65.38%) participants were never drinkers. The mean baseline BMI was 22.02 ± 4.46 kg/m^2^, and the percentages of participants classified as underweight, normal, overweight, and obese were 18.53%, 55.46%, 19.39%, and 6.62%, respectively. The mean PDI, hPDI, and uPDI were 48.71 ± 6.05, 54.09 ± 5.38, and 42.78 ± 6.65 at baseline, respectively. The distribution of baseline covariates by baseline BMI level is shown in Table 1.

**Figure 2:**
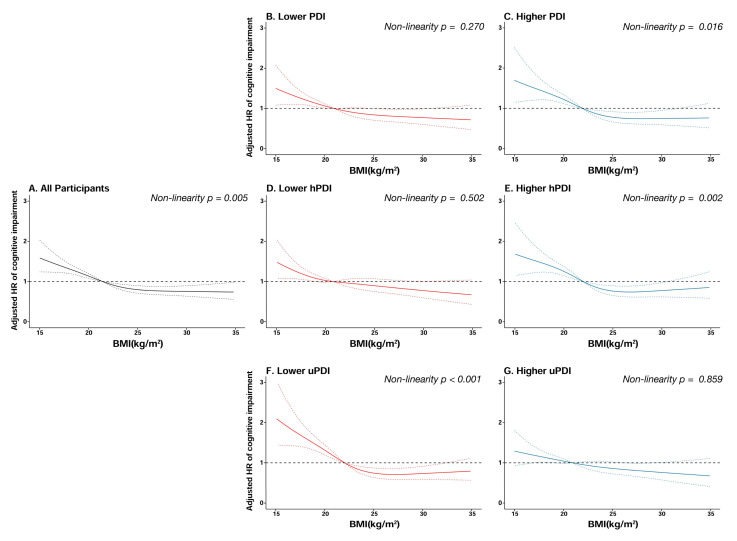
Cubic splines for the associations of baseline BMI with cognitive impairment, stratified by plant-based diet indices. (**A**): all participants; (**B**): lower plant-based diet index (PDI); (**C**): higher plant-based diet index (PDI); (**D**): lower healthful plant-based diet index (hPDI); (**E**): higher healthful plant-based diet index (hPDI); (**F**): lower unhealthful plant-based diet index (uPDI); (**G**): higher unhealthful plant-based diet index (uPDI).

During the 24156 person-years of follow-up, 1077 participants developed cognitive impairment. As shown in Table 2, after multivariable adjustment, as compared with the normal weight group, the HRs of cognitive impairment were 1.42 (95% CI = 1.21–1.66, *p* < 0.001) in the underweight group, 0.79 (95% CI = 0.66–0.95, *p* = 0.010) in the overweight group, and 0.72 (95% CI = 0.54–0.96, *p* = 0.026) in the obese group. Baseline BMI was non-linearly correlated to the risk of cognitive impairment, with a reverse J-shaped relationship (*p* for non-linear trend = 0.005). (See Figure 2.)

After multivariable adjustment, a lower PDI, a lower hPDI, and a higher uPDI were related to an increased risk of cognitive impairment. The HRs of cognitive impairment were 1.32 (95% CI = 1.16–1.50, *p* < 0.001) in the lower PDI group compared with the higher PDI group; the HRs of cognitive impairment were 1.46 (95% CI = 1.29–1.66, *p* < 0.001) in the lower hPDI group as compared with the higher hPDI group, and the HRs of cognitive impairment were 1.21 (95% CI = 1.06–1.38, *p* = 0.004) in the higher uPDI group as compared with the lower uPDI group (Table 3).

**Figure 3:**
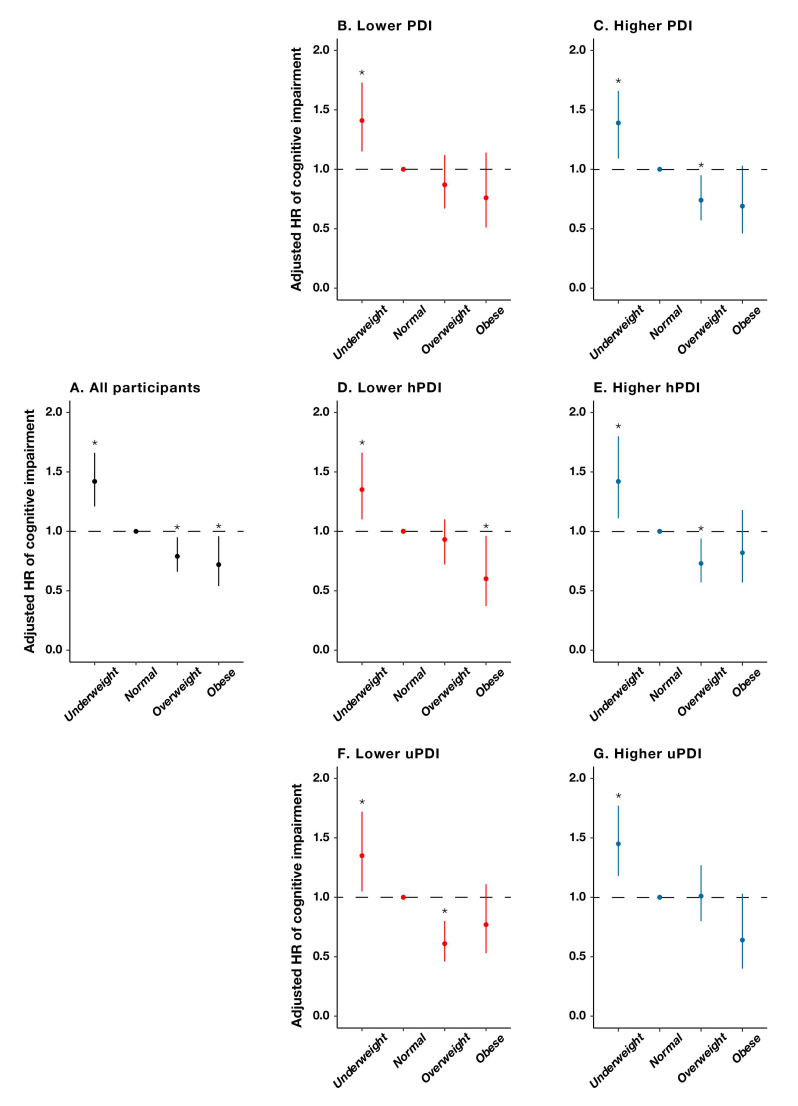
Hazard ratios and 95% CIs for developing cognitive impairment by baseline body-mass-index groups, stratified by plant-based diet indices. *: *p* < 0.05. (**A**): all participants; (**B**): lower plant-based diet index (PDI); (**C**): higher plant-based diet index (PDI); (**D**): lower healthful plant-based diet index (hPDI); (**E**): higher healthful plant-based diet index (hPDI); (**F**): lower unhealthful plant-based diet index (uPDI); (**G**): higher unhealthful plant-based diet index (uPDI).

We observed a significant interaction between baseline BMIs and PDIs, with the corresponding associations of an overweight status being much more pronounced among participants with a higher PDI than those with a lower PDI, among participants with a higher hPDI than those with a lower hPDI, and among participants with a lower uPDI than those with a higher hPDI (Figure 3). Specifically, the protective effect of being overweight on cognitive impairment was attenuated with a 13% (95% CI = 0.67–1.12, *p* = 0.267) decreased risk, which was not significant among those with a lower PDI, in contrast with a 26% (95% CI = 0.57–0.95, *p* = 0.017) decreased risk, which was significant among those with a higher PDI. Similarly, the protective effect of an overweight status on cognitive impairment was attenuated with a 7% (95% CI = 0.72–1.10, *p* = 0.568) non-significant decrease in risk among those with a lower hPDI, in contrast with a 27% (95% CI = 0.57–0.94, *p* = 0.013) significantly decreased risk for those with a higher hPDI. In addition, the protective effect of an overweight status on cognitive impairment was attenuated with a 1% (95% CI = 0.89–1.61, *p* = 0.234) non-significant increase in risk among those with a higher uPDI, in contrast with a 39% (95% CI = 0.46–0.80, *p* < 0.001) significant decrease in risk among those with a lower uPDI (Table 4).

## 4. Discussion

Based on a national, prospective, and community-based cohort, we found that BMI-defined overweight status and obese status were related to decreased risks of cognitive impairment, while an underweight status was related to an increased risk. We also found that lower PDIs, lower hPDIs, and higher uPDIs were associated with increased risks of cognitive impairment. In addition, the protective effect of being overweight on cognitive impairment was more pronounced among participants with higher PDIs than those with lower PDIs, among participants with higher hPDIs than those with lower hPDIs, and among participants with lower uPDIs than those with higher uPDIs. Our results indicated that a lower adherence to an overall and healthful PBD and a higher adherence to an unhealthful PBD may attenuate the protective effect of an overweight status on cognitive impairment.

The relationship of BMI with cognitive function has been reported in numerous studies with inconsistent findings. Some studies found neuroprotective effects for the BMI-defined statuses of overweight and obese in later life [14,15,16,17,18,19,20], while some research reported detrimental neurological effects caused by BMI-defined obesity [6,9,10,11,12]. We found that a reverse J-shaped relationship of BMI with cognitive impairment was identified in the current research, suggesting that the BMI-defined statuses of overweight and obese could be related to a decreased risk of cognitive impairment and that the BMI-defined status of underweight could be related to an increased risk. The aforementioned findings were consistent with those from previous studies targeting a Chinese population [13,21,22,23]. For example, a Chinese cohort study, which included 12,027 individuals 65 years of age and older, found that a BMI-defined overweight status was related to a 16% decreased risk of cognitive impairment [22]. In addition, our findings suggested that a BMI-defined underweight status predicted a higher risk of cognitive impairment in later life. Similarly, the Korean Longitudinal Study of Aging showed that older adults who are underweight may be at a higher risk for cognitive dysfunction [19]. A recent Chinese cohort study of 5156 subjects aged 75 and older reported an increased risk of cognitive impairment significantly associated with a BMI-defined status as underweight [23]. Several pathophysiological mechanisms may help explain our results. First, older individuals with a BMI-defined status as underweight may be experiencing an underlying illness or nutritional deficiencies resulting in a decline in muscle mass, which has been associated with the development of neurodegenerative diseases [47,48]. This is possibly the reason that, in the present study, older individuals with a high BMI demonstrated better cognitive performance as compared with those with a lower BMI. Second, a higher BMI in later life may exert a neuroprotective effect by increasing insulin-growth factor 1 (IGF-1) levels [49], leptin hormone levels [50], and the production of estrogen [51], all of which have been confirmed to be relevant to better cognitive function [52,53]. In addition, a higher leg-fat mass in older adults has been related to improved glucose metabolism [54], which could result in a decreased risk of developing poor cognitive function [55]. Moreover, serum urate, which is positively related to BMI, may slow the progression of neurodegenerative diseases by acting as an antioxidant [56].

There is emerging evidence for the brain-health-promoting effects of several dietary patterns, which promote the high intake of plant-based foods [31,57,58]. Mounting evidence has revealed that PBD patterns can exert neuroprotective effects [26,27]. A cohort study conducted among adults in Singapore reported that participants with higher hPDI scores had a lower risk of cognitive impairment [32]. Recently, a prospective cohort study found that a higher hPDI was related to a slower rate of global cognitive decline, while no association with either PDI or uPDI and cognitive decline was observed [33]. The results of our study show that a lower PDI, a lower hPDI, and a higher uPDI were related to a higher risk of cognitive impairment. The mechanisms underlying this association may be explained by the fact that healthful plant-based foods, such as fruits, vegetables, and nuts, are rich sources of antioxidants and anti-inflammatory nutrients, including polyphenols, flavonoids, antioxidant vitamins, and dietary fiber, which could reduce central nervous system inflammation and oxidative stress [24,25,59,60,61,62,63], ultimately affecting the etiopathogenesis of neurodegenerative diseases [64,65], whereas unhealthful plant-based foods, such as preserved vegetables and added sugars, are high in sodium and sugar, which have been related to decreases in neurological health [66,67]. In addition, unhealthful plant-based foods have previously been linked to increased risks of diabetes and heart disease [29,68], which are also risk factors for decreased neurological health [69,70].

We first demonstrated that a lower adherence to an overall PBD and healthful PBD, and a higher adherence to an unhealthful PBD may attenuate the protective effect of being overweight on cognitive impairment among older adults. This might be because a healthful PBD could reduce inflammation and oxidative stress in the central nervous system as induced by an unfavorable weight status [63]. More studies are needed to explore the moderating role of three plant-based diets in this relationship between BMI and cognitive function so as to elucidate this mechanism.

To our knowledge, we are among the first to assess whether PBD patterns, using the PDI, hPDI, and uPDI, modify the relationship between BMI and cognitive function. In addition, our research is based on a nationally representative sample of older Chinese adults, which facilitates the generalization of our findings. There are also some limitations to the study. First, it should be emphasized that our findings were based on a single measurement of the BMI and diet at baseline, which may not accurately reflect the long-term status. Second, diet was assessed using a simple FFQ without information on portion sizes; hence, we cannot calculate and adjust for total energy intake. In addition, dietary assessment via FFQ may have been subject to recall bias. Third, detailed information for several food items (e.g., potatoes, honey, and berries) was not available in the FFQ in the CLHLS. Further research with more-detailed dietary assessments is required to validate the observed findings. Fourth, the contribution of dietary supplements was not considered in the present study, which could have caused a bias in our results. Fifth, residual, unknown confounding factors cannot be entirely ruled out. All included participants were from China, which limits the extrapolation of our conclusions to other nationalities and ethnic groups. Sixth, given the observational study design, no causal association can be proved.

## 5. Conclusions

Based on a national, community-based, longitudinal prospective study in China, we found that BMI-defined statuses of overweight and obese were related to a decreased risk of cognitive impairment, while an underweight status was related to increased risk. Lower PDI, lower hPDI, and higher uPDI were associated with an increased risk of cognitive impairment. Furthermore, we first demonstrated that a lower adherence to an overall and a healthful PBD and a higher adherence to an unhealthful PBD may attenuate the protective effect of being overweight on cognitive impairment. Our findings are informative in facilitating the development of tailored body-weight-management and dietary recommendations for preventing cognitive impairment in an elderly population.

## Figures and Tables

**Table 1:** Characteristics of the study population at baseline.

Characteristics	Total	Underweight	Normal	Overweight	Obese	*p* Value
*N*	4792	888	2658	929	317	
BMI (kg/m^2^) *	22.02 ± 4.46	16.98 ± 1.28	21.20 ± 1.52	25.58 ± 1.12	32.53 ± 6.94	<0.001
PDI score *	48.71 ± 6.05	47.02 ± 6.34	48.69 ± 6.02	49.90 ± 5.43	50.12 ± 5.98	<0.001
hPDI score *	54.09 ± 5.38	52.56 ± 5.49	54.06 ± 5.40	55.32 ± 4.83	55.07 ± 5.31	<0.001
uPDI score *	42.78 ± 6.65	44.43 ± 6.53	42.87 ± 6.55	41.50 ± 6.58	41.18 ± 6.95	<0.001
Age, years *	80.70 ± 9.58	84.38 ± 9.87	80.71 ± 9.49	78.11 ± 8.76	77.91 ± 8.37	<0.001
Sex, male **	2425 (50.61)	390 (43.92)	1447 (54.44)	466 (50.16)	122 (38.49)	<0.001
Residence **						<0.001
City	782 (16.32)	78 (8.78)	400 (15.05)	224 (24.11)	80 (25.24)	
Town	1517 (31.66)	261 (29.39)	861 (32.39)	287 (30.89)	108 (34.07)	
Rural	2493 (52.02)	549 (61.82)	1397 (52.56)	418 (44.99)	129 (40.69)	
Illiterate **	2339 (48.81)	508 (57.21)	1280 (48.16)	406 (43.70)	145 (45.74)	<0.001
Financial independence **	1157 (24.14)	114 (12.84)	595 (22.39)	324 (34.88)	124 (39.12)	<0.001
With regular exercise **	1997 (41.67)	301 (33.90)	1090 (41.01)	439 (47.26)	147 (46.37)	<0.001
Smoking status **						<0.001
Never smoker	2972 (62.02)	560 (63.06)	1571 (59.10)	604 (65.02)	237 (74.76)	
Former smoker	772 (16.11)	122 (13.74)	460 (17.31)	153 (16.47)	37 (11.67)	
Current smoker	1048 (21.87)	206 (23.20)	627 (23.59)	172 (18.51)	43 (13.56)	
Alcohol consumption **						<0.001
Never drinker	3133 (65.38)	612 (68.92)	1676 (63.05)	616 (66.31)	229 (72.24)	
Former drinker	681 (14.21)	97 (10.92)	403 (15.16)	134 (14.42)	47 (14.83)	
Current drinker	978 (20.41)	179 (20.16)	579 (21.78)	179 (19.27)	41 (12.93)	
Occupation **						0.156
Professional and technical personnel	201 (4.19)	18 (2.03)	111 (4.18)	55 (5.92)	17 (5.36)	
Governmental, institutional, or managerial personnel	165 (3.44)	13 (1.46)	78 (2.93)	52 (5.60)	22 (6.94)	
Commercial, service, or industrial worker	578 (12.06)	56 (6.31)	300 (11.29)	161 (17.33)	61 (19.24)	
Self-employed	81 (1.69)	12 (1.35)	42 (1.58)	22 (2.37)	5 (1.58)	
Agricultural, forestry, animal husbandry, or fishery worker	2972 (62.02)	638 (71.85)	1692 (63.66)	482 (51.88)	160 (50.47)	
Houseworker	213 (4.44)	50 (5.63)	101 (3.80)	47 (5.06)	15 (4.73)	
Military personnel	32 (0.67)	3 (0.34)	22 (0.83)	5 (0.54)	2 (0.63)	
Never worked	16 (0.33)	2 (0.23)	9 (0.34)	3 (0.32)	2 (0.63)	
Others	534 (11.14)	96 (10.81)	303 (11.40)	102 (10.98)	33 (10.41)	
Disease score ***	1 (1)	1 (1)	1 (1)	1 (1)	1 (1)	<0.001
Hypertension **	1480 (30.88)	177 (19.93)	749 (28.18)	394 (42.41)	160 (50.47)	<0.001
Diabetes **	230 (4.80)	16 (1.80)	95 (3.57)	83 (8.93)	36 (11.36)	<0.001
Heart diseases **	332 (6.93)	42 (4.73)	174 (6.55)	90 (9.69)	26 (8.20)	<0.001
Stroke **	342 (7.14)	44 (4.95)	44 (1.66)	109 (11.73)	189 (59.62)	0.001
Cancer **	27 (0.56)	4 (0.45)	13 (0.49)	10 (1.08)	0 (0.00)	0.093
Respiratory disease **	534 (11.14)	124 (13.96)	281 (10.57)	90 (9.69)	39 (12.30)	0.008

Abbreviations: BMI: body mass index; PDI: plant-based diet index; hPDI: healthful plant-based diet index; uPDI: unhealthful plant-based diet index. *: mean (standard deviation) was reported; **: Number (%) was reported; ***: median (interquartile range) was reported.

**Table 2:** Association of baseline BMI with incidence of cognitive impairment risk.

	Events	Participants	Person-Years	HR (95% CI) ^a^	*p* Value
Underweight	263	888	4072	1.42 (1.21–1.66)	<0.001
Normal	579	2658	13,498	1.00	
Overweight	172	929	4891	0.79 (0.66–0.95)	0.010
Obese	63	317	1695	0.72 (0.54–0.96)	0.026

HR: hazard ratio; CI: confidence interval. ^a^: Adjusted for sex, age, residence, education, occupation, smoking status, alcohol consumption, regular exercise, financial independence, and health conditions.

**Table 3:** Associations of baseline plant-based diet indices with cognitive impairment risk.

	Events	Participants	Person-Years	HR (95% CI) ^a^	*p* Value
Stratified by PDI					
Lower PDI	594	2274	11,330	1.32 (1.16–1.50)	<0.001
Higher PDI	483	2518	12,826	1.00	
Stratified by hPDI					
Lower hPDI	561	2081	10,295	1.46 (1.29–1.66)	<0.001
Higher hPDI	516	2711	13,861	1.00	
Stratified by uPDI					
Lower uPDI	480	2462	12,490	1.00	
Higher uPDI	597	2330	11,666	1.21 (1.06–1.38)	0.004

HR: hazard ratio; CI: confidence interval; PDI: plant-based dietary index; hPDI: healthful plant-based dietary index; uPDI: unhealthful plant-based dietary index. ^a^: Adjusted for sex, age, residence, education, occupation, smoking status, alcohol consumption, regular exercise, financial independence, and health conditions.

**Table 4:** Associations of baseline BMIs with cognitive impairment risk, stratified by plant-based diet indices.

	Events	Participants	Person-Years	HR (95% CI) ^a^	*p* Value
Stratified by PDI					
Lower PDI					
Underweight	163	514	2346	1.41 (1.15–1.73)	0.001
Normal	314	1263	6373	1.00	
Overweight	88	370	1966	0.87 (0.67–1.12)	0.267
Obese	29	127	645	0.76 (0.51–1.14)	0.188
Higher PDI					
Underweight	100	374	1726	1.39 (1.09–1.77)	0.007
Normal	265	1395	7125	1.00	
Overweight	84	559	2925	0.74 (0.57–0.95)	0.017
Obese	34	190	1050	0.69 (0.46–1.03)	0.068
Stratified by hPDI					
Lower hPDI					
Underweight	163	487	2221	1.35 (1.10–1.66)	0.004
Normal	299	1172	5840	1.00	
Overweight	77	310	1630	0.93 (0.72–1.10)	0.568
Obese	22	112	604	0.60 (0.37–0.96)	0.035
Higher hPDI					
Underweight	100	401	1851	1.42 (1.11–1.80)	0.005
Normal	280	1486	7658	1.00	
Overweight	95	619	3261	0.73 (0.57 –0.94)	0.013
Obese	41	205	1091	0.82 (0.57–1.18)	0.284
Stratified by uPDI					
Lower uPDI					
Underweight	98	372	1692	1.35 (1.05–1.72)	0.017
Normal	271	1348	6860	1.00	
Overweight	71	539	2837	0.61 (0.46–0.80)	<0.001
Obese	40	203	1101	0.77 (0.53–1.11)	0.158
Higher uPDI					
Underweight	165	516	2380	1.45 (1.18–1.77)	<0.001
Normal	308	1310	6638	1.00	
Overweight	101	390	2054	1.01 (0.80–1.27)	0.955
Obese	23	114	594	0.64 (0.40–1.03)	0.066

HR: hazard ratio; CI: confidence interval; PDI: plant-based diet index; hPDI: healthful plant-based diet index; uPDI: unhealthful plant-based diet index. ^a^: Adjusted for sex, age, residence, education, occupation, smoking status, alcohol consumption, regular exercise, financial independence, and health conditions.

## Data Availability

The data of this study are available to researchers upon reasonable request to corresponding author.

## Appendix A

**Table A1:** Plant-based diet index scoring.

Food Category		Food Groups	Frequency	PDI	hPDI	uPDI
Plant-based food	Healthful	Whole grain	Yes	5	5	1
			No	1	1	5
		Vegetable oils	Yes	5	5	1
			No	1	1	5
		Fresh fruits	Almost everyday	5	5	1
			Quite often	4	4	2
			Occasionally	2	2	4
			Rarely or never	1	1	5
		Fresh vegetables	Almost everyday	5	5	1
			Quite often	4	4	2
			Occasionally	2	2	4
			Rarely or never	1	1	5
		Legumes	Almost everyday	5	5	1
			≥1 time/week	4	4	2
			≥1 time/month	3	3	3
			Occasionally	2	2	4
			Rarely or never	1	1	5
		Garlic	Almost everyday	5	5	1
			≥1 time/week	4	4	2
			≥1 time/month	3	3	3
			Occasionally	2	2	4
			Rarely or never	1	1	5
		Nuts	Almost everyday	5	5	1
			≥1 time/week	4	4	2
			≥1 time/month	3	3	3
			Occasionally	2	2	4
			Rarely or never	1	1	5
		Tea	Almost everyday	5	5	1
			≥1 time/week	4	4	2
			≥1 time/month	3	3	3
			Occasionally	2	2	4
			Rarely or never	1	1	5
	Unhealthful	Refined grains	Yes	5	1	5
			No	1	5	1
		Sugar (white granulated sugar or candies)	Almost everyday	5	1	5
			≥1 time/week	4	2	4
			≥1 time/month	3	3	3
			Occasionally	2	4	2
			Rarely or never	1	5	1
		Preserved vegetables	Almost everyday	5	1	5
			≥1 time/week	4	2	4
			≥1 time/month	3	3	3
			Occasionally	2	4	2
			Rarely or never	1	5	1
Animal-based food		Animal fat	Yes	1	1	1
			No	5	5	5
		Meat	Almost everyday	1	1	1
			≥1 time/week	2	2	2
			≥1 time/month	3	3	3
			Occasionally	4	4	4
			Rarely or never	5	5	5
		Fish	Almost everyday	1	1	1
			≥1 time/week	2	2	2
			≥1 time/month	3	3	3
			Occasionally	4	4	4
			Rarely or never	5	5	5
		Eggs	Almost everyday	1	1	1
			≥1 time/month	3	3	3
			Occasionally	4	4	4
			Rarely or never	5	5	5
		Dairy products	Almost everyday	1	1	1
			≥1 time/week	2	2	2
			≥1 time/month	3	3	3
			Occasionally	4	4	4
			Rarely or never	5	5	5

Abbreviations: PDI: plant-based diet index; hPDI: healthful plant-based diet index; uPDI: unhealthful plant-based diet index.
